# Search for germline gene variants in colorectal cancer families presenting with multiple primary colorectal cancers

**DOI:** 10.1002/ijc.35283

**Published:** 2024-12-10

**Authors:** Asta Försti, Filip Ambrozkiewicz, Magdalena Marciniak, Jan Lubinski, Kari Hemminki

**Affiliations:** ^1^ Hopp Children's Cancer Center (KiTZ) Heidelberg Germany; ^2^ Division of Pediatric Neurooncology, German Cancer Research Center (DKFZ) German Cancer Consortium (DKTK) Heidelberg Germany; ^3^ Biomedical Center, Faculty of Medicine Charles University Pilsen Pilsen Czech Republic; ^4^ Department of Genetics and Pathology, International Hereditary Cancer Center Pomeranian Medical University in Szczecin Szczecin Poland; ^5^ Division of Cancer Epidemiology German Cancer Research Center (DKFZ) Heidelberg Germany

**Keywords:** familial colorectal cancer, germline variant, multiple primaries, whole‐exome sequencing

## Abstract

A double primary colorectal cancer (CRC) in a familial setting signals a high risk of CRC. In order to identify novel CRC susceptibility genes, we whole‐exome sequenced germline DNA from nine persons with a double primary CRC and a family history of CRC. The detected variants were processed by bioinformatics filtering and prioritization, including STRING protein–protein interaction and pathway analysis. A total of 150 missense, 19 stop‐gain, 22 frameshift and 13 canonical splice site variants fulfilled our filtering criteria. The STRING analysis identified 20 DNA repair/cell cycle proteins as the main cluster, related to genes *CHEK2*, *EXO1*, *FAAP24*, *FANCI*, *MCPH1*, *POLL*, *PRC1*, *RECQL*, *RECQL5*, *RRM2*, *SHCBP1*, *SMC2*, *XRCC1*, in addition to *CDK18*, *ENDOV*, *ZW10* and the known mismatch repair genes. Another STRING network included extracellular matrix genes and TGFβ signaling genes. In the nine whole‐exome sequenced patients, eight harbored at least two candidate DNA repair/cell cycle/TGFβ signaling gene variants. The number of families is too small to provide evidence for individual variants but, considering the known role of DNA repair/cell cycle genes in CRC, the clustering of multiple deleterious variants in the present families suggests that these, perhaps jointly, contributed to CRC development in these families.

## Graphical abstract

What's new?

A double primary colorectal cancer case in a familial setting signals a high risk of colorectal cancer. Here, the authors performed whole‐exome sequencing in nine patients with a double primary cancer and a family history of colorectal cancer to identify novel predisposition genes. Using bioinformatics filtering and prioritization, they found 204 potentially deleterious variants. STRING protein–protein interaction network and pathway analysis highlighted the importance of the DNA repair, cell cycle, and TGF‐β signaling gene variants. The clustering of multiple deleterious variants in each patient suggests a joint effect on their family's colorectal cancer predisposition.

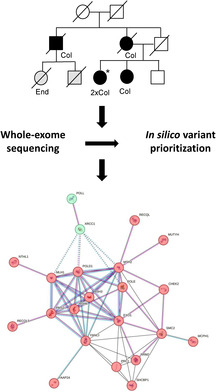

## 1 INTRODUCTION

Germline genetics of colorectal cancer (CRC) is dominated by mismatch repair (MMR) gene defects.^1^ One of the earliest whole‐exome sequencing studies on familial early‐onset CRC found that MMR gene mutations accounted for 76% of all identified mutations in the known CRC predisposition genes, *APC* for 11% and *MUTYH* for 8%.^2^ Age of onset of CRC in MMR gene mutation carriers has been reported from the prospective Lynch syndrome database, ranging from 50 years for *MLH1*, 56–57 years for *MSH2*, over 60 years for *MSH6* and over 70 years for the rare *PMS2*.^3^ *POLE/POLD1* and *NTHL1* are among the genes that were confirmed as predisposition genes more recently.^1^ Many of these genes are related to DNA repair (e.g., *POLE/POLD1*). Some are related to TGFβ/BMP pathway (e.g., *BMPR1A* and *SMAD4*). Currently, several whole‐exome and whole‐genome approaches in CRC families are going on, with the aim to identify novel high‐to‐moderate penetrance CRC predisposition genes.^1^ Previously, we undertook a linkage study in Mendelian type of families.^4^ Family members concordant for CRC were analyzed by whole‐exome or whole‐genome sequencing and a number of promising candidate genes were detected in diverse families. These included *APC downregulated 1* (*APCDD1*), *histone deacetylase 5* (*HDAC5*), and intestinal mucus barrier‐regulating genes, *CYBA* and *TRPM4*.^5^, ^6^ Although the genes appeared mechanistically plausible, the variants were rare and await independent confirmation in other CRC families. Early‐onset CRC (diagnosed before age, e.g., 50 years) has recently attracted interest for germline studies but the spectrum of detected genes is dominated by the genes found even in older patients, and some 10% of patients are carriers of known CRC predisposing genes and over 2% carry variants known to predispose to other familial cancers.^7^ Of course, we need to keep in mind the scope for low‐risk polygenic inheritance.^8^, ^9^

For the present study, we modified our study strategy by considering CRC families where some affected family members had two primary CRCs. The reasoning with this was that while familial risk for CRC is 1.70, when one first‐degree family member has been diagnosed with CRC, it increases to 2.76, when two or more family members have been diagnosed.^10^ The risk is only slightly lower (2.67) when apparent Lynch syndrome families have been removed.^11^ Risk for double primary CRCs is high (about 3 on top of the familial risk, thus combined >5) in persons with a personal and family history of CRC. Thus, the likelihood of finding novel susceptibility genes should be high in CRC families also presenting with double primary CRC.^12^ We selected nine Polish CRC families with a prominent family history of CRC: The total number of CRCs in these families was 39. Two of these families were sequenced in our previous projects.^5^, ^6^ From each of these families, the family member with two primary CRCs was subjected to whole‐exome sequencing (WES) for identification of novel CRC predisposition genes.

## 2 MATERIALS AND METHODS

### 2.1 Population

Altogether 28 Polish families with at least one family member diagnosed with two or more CRCs as an index case were identified at the International Hereditary Cancer Center in Szczecin, Poland (Table S1). Two of these families were reported in our previous publications.^5^, ^6^ For this study we included nine families, including the two previously reported ones, from which germline DNA from a blood sample of the index case, with double primary CRC and family history of CRC, was submitted to WES (Figure S1). Each of the selected families had a prominent family history of CRC, with the index case diagnosed with two CRCs, at least one other family member affected by CRC and earliest age at diagnosis ranging from 28 to 64 years.

### 2.2 Whole‐exome sequencing

Library preparation and WES of the newly identified seven index cases was performed by BGI Genomics using Agilent V6‐based sequencing followed by the standard bioinformatics pipeline provided by BGI Genomics (https://www.bgi.com/global). Reads were aligned to the human reference genome (NCBI build GRCh38) using BWA software. BGI used GATK for single nucleotide variant (SNV) calling and annotation was based on the human RefGene database. For SNV validation and comparison, dbSNP database, 1000 Genomes Project database, publicly available exome databases (ESP, ExAC), ENCODE, ClinVar and GWAS data were used by the BGI. Functionality and conservation prediction of SNVs in the BGI pipeline was performed with the following in silico tools: SIFT, Polyphen‐2, Mutation Assessor, LRT, MutationTaster, FATHMM, MetaSVM, MetaLR, VEST3, CADD, GERP++, phyloP, phastCons and SiPhy. In the BGI pipeline, Small Insertion/Deletion variants (InDels) were called using GATK followed by the annotation to the human RefGene database and validation and comparison with the same databases as for SNVs. The sequencing coverage and quality statistics for each sample were provided by BGI and are summarized in Table S2.

### 2.3 Variant annotation and filtering

We annotated the processed list of the SNVs and small InDels further using the Ensembl Variant Effect Prediction (VEP) tool.^13^ We included only variants affecting the Ensembl canonical/protein coding transcript. Minor allele frequency (MAF) filter of 0.1% was used with respect to gnomAD_exomes_AF, gnomAD_exomes_NFE_AF and gnomAD_exomes_POPMAX_AF data to remove common variants (gnomAD v.4.0.0; https://gnomad.broadinstitute.org). Rare SNVs and InDels ranking within the top 1% of potentially deleterious variants in the human genome were selected using the Combined Annotation Dependent Depletion (CADD) tool; a scaled PHRED‐like CADD score greater than 20 was applied.^14^

### 2.4 Variant prioritization: Missense variants

Four steps were used to identify the most likely missense variants as candidates for novel CRC susceptibility genes. Firstly, the variants were screened for their potential deleteriousness by using nine different prediction tools: Sorting Intolerant from Tolerant (SIFT),^15^ Polymorphism Phenotyping version 2 (PolyPhen‐2),^16^ Log ratio test (LRT),^17^ MutationTaster,^18^ Mutation Assessor,^19^ Functional Analysis Through Hidden Markov Models (FATHMM),^20^ MetaSVM,^21^ MetaLR^21^ and Protein Variation Effect Analyzer (PROVEAN).^22^ Variants predicted to be deleterious by over 50% of these tools were selected. Secondly, the variants should be located at an evolutionary conserved position, which was evaluated by Genomic Evolutionary Rate Profiling (GERP > 2.0),^23^ PhastCons (>0.3)^24^ and Phylogenetic *p*‐value (PhyloP ≥ 3.0),^25^ with an inclusion cutoff of at least two positive predictions. Thirdly, based on the assumption that variants within genes intolerant to variation are likely to be deleterious, we report the *Z*‐score, developed by the gnomAD consortium for missense variants.^26^ Positive *Z*‐scores indicate increased constraint, that is, intolerance to variation. Fourthly, we report the AlphaMissense^27^ and REVEL^28^ scores for variants that passed the MAF < 0.001, CADD > 20, deleteriousness and conservation criteria of our pipeline.

### 2.5 Loss‐of‐function variant analysis

Stop‐gain, frameshift and splice‐site variants affecting the canonical splice sites were considered when the CADD score criterion of >20 was met. Variants affecting the last exon of the gene were excluded from further analyses, all other variants are reported. It is well known that also healthy people carry genetic variants predicted to cause loss‐of function (LoF).^29^ In order to discriminate pathogenic and neutral variants, we used MutPred2 (http://mutpred.mutdb.org).^30^ For each variant, it returns a score between zero and one; higher scores denote variants that are more likely to be pathogenic; a conservative threshold score of 0.50 at 5% false‐positive rate is recommended. MutPred2 also shows structural and functional mechanisms that are impacted in the affected region of the protein, accompanied by significant prior‐corrected *p*‐values. Splice site variants were analyzed by using SpliceAI^31^ and MMSplice^32^ within the Ensembl VEP tool. The SpliceAI Δ score ≥0.5 indicates a confidently predicted cryptic splice variant and Δ score ≥0.8 indicates a high‐scoring predicted cryptic splice variant. The MMSplice score (absolute value) >2 indicates a high confidence level prediction and >1.5 medium level confidence. For LoF variants we report the LOEUF (loss‐of‐function observed/expected upper bound fraction) score from gnomAD which reflects the gene constraint; gnomAD recommends to use LOEUF <0.6 as an indication of increased gene constraint, if a cut‐off is needed, although many tumor suppressor genes, such as the MMR genes have a higher LOEUF.

### 2.6 Network analysis with STRING

Protein–protein interactions within a biologically relevant pathway may give information about the importance of the identified genes in CRC development. We used the in silico tool STRING^33^ to investigate the interactions between the proteins encoded by the prioritized genes and 16 well‐known CRC predisposition genes^34^ and functional enrichments within the created network. To create the network we used experiments, databases and co‐expression as active interaction sources with a minimum required interaction score 0.400. From the interaction plot of all corresponding proteins we selected the largest networks to investigate specific functional enrichments within these networks. In the enrichment analyses we focused on Biological Processes within Gene Ontology (GO), Kyoto Encyclopedia of Genes and Genomes (KEGG) and Reactome pathways. All statistical analyses are based on the STRING software tool. There, the PPI enrichment analysis is based on the input data compared to the random list of proteins. Such an enrichment indicates that the proteins are at least partially biologically connected, as a group. To calculate the false discovery rate (FRD), STRING uses the Benjamini‐Hochberg procedure. Further information of genes, proteins and their function were collected using GeneCards (https://www.genecards.org) and UniProt (https://www.uniprot.org). PubMed (https://pubmed.ncbi.nlm.nih.gov) was used to search for information about the relationship between the genes with cancer, especially in the context of CRC.

## 3 RESULTS

**FIGURE 1:**
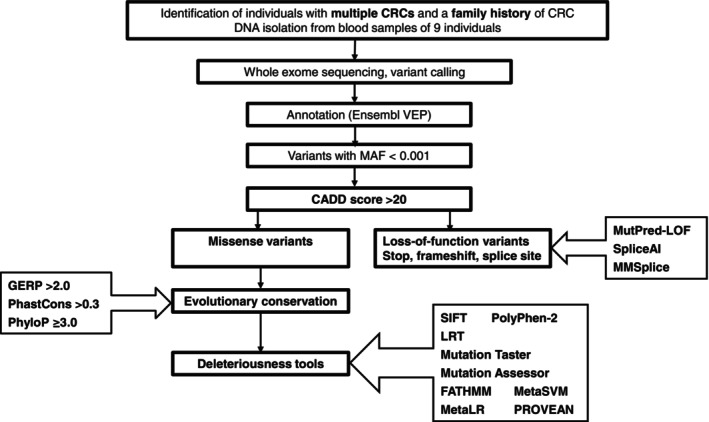
Pipeline for prioritization of the germline variants identified in cancer patients with personal and family history of CRC.

We identified nine families with a strong family history of CRC strengthened by a family member diagnosed with two primary CRCs as the index case whose DNA was whole‐exome sequenced (Figure S1). We screened the WES data for known CRC predisposition gene variants and found no high‐penetrance alleles. We used the pipeline shown in Figure 1 to prioritize the variants and to identify the corresponding genes as candidates for novel CRC susceptibility genes. We identified altogether 204 variants including 150 missense, 19 stop‐gain, 22 frameshift and 13 canonical splice site variants that fulfilled our filtering criteria (Table S3).

In the index case of family F6 we identified an already known moderate‐penetrance variant in *CHEK2*, I157T.^35^, ^36^ The family has a very strong family history of CRC: the index case was diagnosed for her 1st CRC at the age of 28 years and for her 2nd CRC at the age of 53 years. Her sister had developed a CRC at the age of 43 years and her father at the age of 63 years. Also, the uncle from the father's side had a CRC diagnosis. This family had previously been screened using denaturing high‐performance liquid chromatography (DHPLC). Among the other 19 families considered in the beginning for our study, two index cases with double primary CRC had been found to carry the *CHEK2* I157T variant using the next‐generation sequencing HiRisk panel (Table S1). In these families the family history was less prominent: in family 5, only the index case had CRC at the age of 46 and 64 years; in family 41, in addition to the index case (CRC at age 39 and 44 years) the father had CRC at the age 54 years.

In addition to the *CHEK2* I157T variant in family F6, we also identified several other interesting variants in the same family, including a frameshift variant in *SMAD4*, which predisposes to juvenile polyposis syndrome (Table S3).^1^ The presence of the variant was confirmed by Sanger sequencing. The variant Gly255SerfsTer80 leads to a truncated protein at position 335 within the MH2 domain. The variant is not reported in ClinVar^37^ or Leiden Open Variation Database (LOVD) v.3.0 Build 29b.^38^ However, in ClinVar nearly all frameshift variants are classified as pathogenic in juvenile polyposis syndrome and in LOVD variants causing a protein truncation around position 335 are classified as pathogenic.

The detected 204 variants affected 203 genes; only for *DMBT1* two variants were found, a missense variant Ala636Thr in family F6 and a stop‐gain variant Arg614Ter in family F34 (Table S3). The latter variant had a MutPred2 LOF‐score of 0.5 and it was predicted to affect manganese binding, disulfide linkage, RNA binding, cadmium binding and iron binding. DMBT1 has been implicated in immune defense and in epithelial differentiation.^39^ ClinVar and LOVD do not report any variants creating a stop codon, and 2 InDels variants at amino acid positions 584 and 585 reported by ClinVar are classified as likely benign.

**FIGURE 2:**
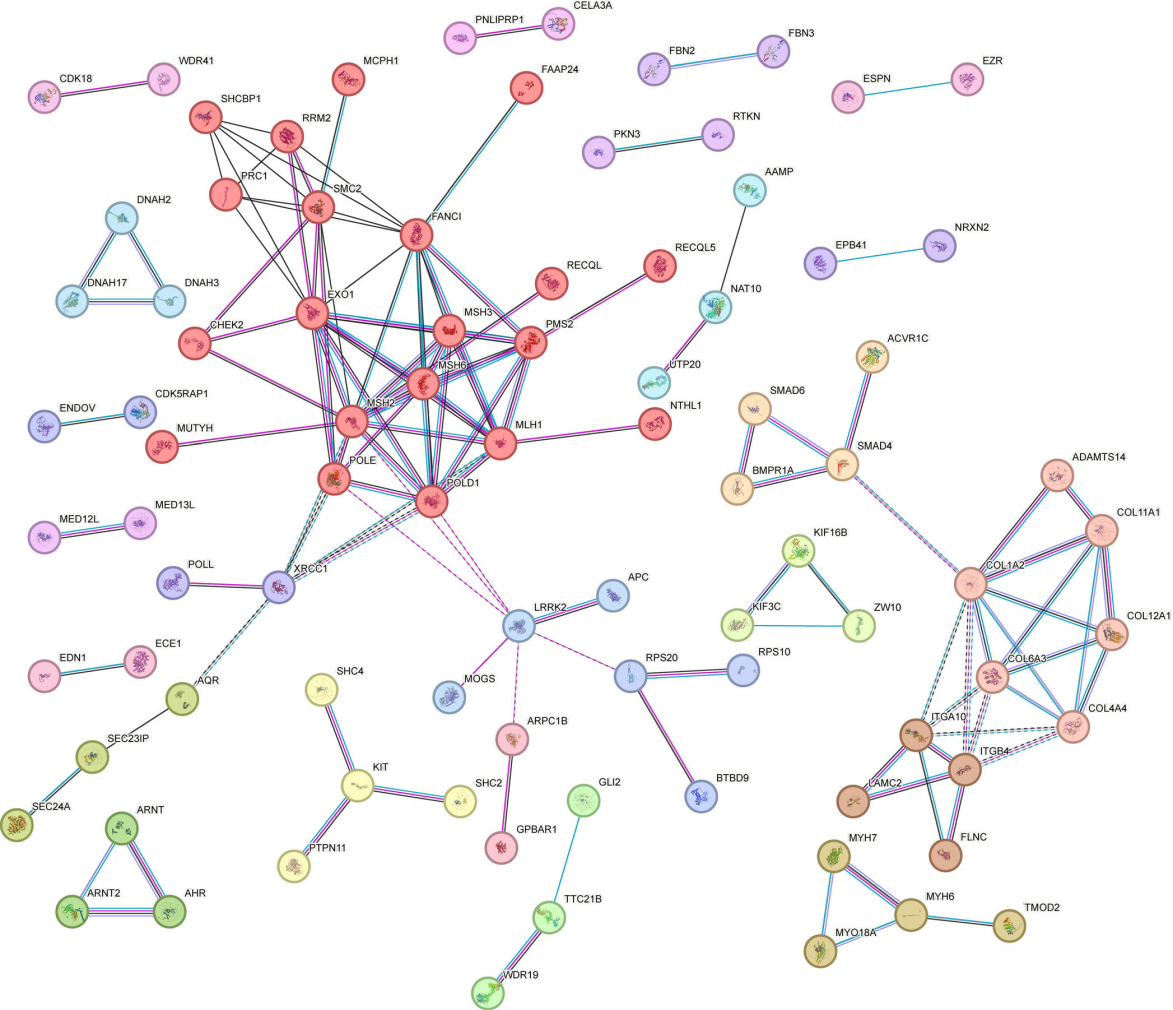
Protein–protein interaction network of proteins encoded by the prioritized genes. Only connected nodes are shown. Each cluster identified by STRING is shown in its own color. Purple line between the nodes indicates experimental evidence of interaction between the proteins, light blue line indicates database evidence and black line co‐expression evidence.

We combined the 203 genes from our study and the 16 well‐known CRC predisposition genes described in Valle et al.^34^ into a single list and used the STRING protein–protein interaction (PPI) network and pathway analysis to identify proteins that interact with each other and are involved in pathways relevant to CRC development. Altogether 88 proteins interacted with at least one other protein (PPI enrichment *p*‐value 2.05 × 10^−5^; Figure 2); 48 of these genes were involved in the GO Biological Process “Cellular component organization or biogenesis” (FDR 0.00035).

The largest network contained an interaction cluster of 20 proteins involved in GO Biological Process “DNA repair” (FDR 0.0021) and “Cell cycle” (FDR 0.0021) (Table 1). The known CRC susceptibility genes related to “DNA repair,” *MLH1*, *MSH2*, *MSH3*, *MSH6*, *MUTYH*, *NTHL1*, *PMS2*, *POLD1* and *POLE*, formed the core of the network, to which the genes *CHEK2*, *EXO1*, *FAAP24*, *FANCI*, *RECQL* and *RECQL5* from our list were connected. A cluster of two genes, *POLL* and *XRCC1*, from GO “Base‐excision repair” was also connected to this cluster. Two of the genes, *POLL* and *RECQL* (stop‐gain) were present in family F7 and three, *EXO1* (stop‐gain), *RECQL5* and *XRCC1* in family F12_S2. The *RECQL* variant had a MutPred LOF‐score of 0.61 and it was predicted to affect magnesium binding and amidation. The *EXO1* variant had a MutPred LOF‐score of 0.5. Details of the identified variants in genes in this network are shown in Table 2.

**TABLE 1:** Protein clusters identified by the STRING analysis of known CRC predisposition genes together with the prioritized genes in our study.

Cluster ID^a^	Gene count	Protein names	Pathway
Cluster 1	20	CHEK2, EXO1, FAAP24, FANCI, MCPH1, MLH1, MSH2, MSH3, MSH6, MUTYH, NTHL1, PMS2, POLD1, POLE, PRC1, RECQL, RECQL5, RRM2, SHCBP1, SMC2	Known CRC susceptibility genes: MLH1, MSH2, MSH3, MSH6, MUTYH, NTHL1, PMS2, POLD1, POLE; Base excision repair: MUTYH, NTHL1, POLD1, POLE; Mismatch repair: EXO1, MLH1, MSH2, MSH3, MSH6, MUTYH, PMS2; Other DNA repair genes: CHEK2, FAAP24, FANCI, RECQL, RECQL5; Mitotic cell cycle: CHEK2, MCPH1, MSH2, POLE, PRC1, RECQL5, SMC2; Other cell cycle genes: EXO1, FANCI, MLH1, MSH6
Cluster 2	6	ADAMTS14, COL11A1, COL12A1, COL1A2, COL4A4, COL6A3	ECM‐receptor interaction: COL1A2, COL4A4, COL6A3; Degradation of ECM: COL11A1, COL12A1, COL1A2, COL4A4, COL6A3; Focal adhesion: COL1A2, COL4A4, COL6A3
Cluster 3	4	FLNC, ITGA10, ITGB4, LAMC2	ECM‐receptor interaction: ITGA10, ITGB4, LAMC2; Degradation of ECM: LAMC2; Focal adhesion: FLNC, ITGA10, ITGB4, LAMC2
Cluster 4	4	ACVR1C, BMPR1A, SMAD4, SMAD6	Known CRC susceptibility genes: BMPR1A, SMAD4; TGFβ signaling pathway: ACVR1C, BMPR1A, SMAD4, SMAD6
Cluster 16	2	POLL, XRCC1	Base excision repair: POLL, XRCC1

*Note*: Only clusters related to DNA repair, cell cycle, extracellular matrix (ECM) organization, focal adhesion and TGFβ signaling pathway are shown.

^a^ Cluster ID refers to the cluster ID given by the STRING analysis.

**TABLE 2:** Overview of the variants and related genes and proteins from the main STRING protein–protein interaction clusters.

Family_ID	Gene	Gene name	Chrom_Pos_Ref_Alt	Ensemble transcript; HGVSc	HGVSp	CADD_phred	Deleteriousness score n/9	gnomAD *Z*‐score or LOEUF score^a^	MutPred2_LOF score	MutPred2 mechanisms	Protein function
DNA repair and cell cycle											
F6	CHEK2	Checkpoint Kinase 2	22_28725099_T_C	ENST00000404276.6:c.470T>C	p.Ile157Thr^d^	23.5	5.5	−0.02			Cell cycle; DNA repair
F7	POLL	DNA Polymerase Lambda	10_101583527_G_A	ENST00000370162.8:c.1046C>T	p.Ala349Val^d^	29.1	8	1.61			Base excision repair
F7	RECQL	RecQ like helicase	12_21486541_G_A	ENST00000444129.7:c.439C>T	Gln147Ter	38		1.03	0.61	Magnesium binding (*p* = 0.036); Amidation (*p* = 0.040)	DNA repair
F7	SHCBP1	SHC binding and spindle associated 1	16_46604439_G_A	ENST00000303383.8:c.712C>T	Arg238Ter	36		1.02	0.4	NA	Cell proliferation, growth and differentiation
F8	PRC1	Protein Regulator Of Cytokinesis 1	15_90979162_A_C	ENST00000394249.8:c.1103T>G	p.Phe368Cys^d^	32	5.5	0.76			Cell cycle; mitosis
F29	CDK18	Cyclin Dependent Kinase 18	1_205530350_G_A	ENST00000429964.7:c.1312+1G>A^b^		34		1.08			Cell cycle; mitosis
F29	RRM2	Ribonucleotide Reductase Regulatory Subunit M2	2_10128903_C_T	ENST00000304567.10:c.854C>T	p.Ser285Leu	22.9	7	2.1			DNA synthesis
F34	FAAP24	FA Core Complex Associated Protein 24	19_32976560_C_T	ENST00000588258.6:c.526C>T	p.Leu176Phe	26.5	5.5	0.97			DNA repair
F34	SMC2	Structural Maintenance Of Chromosomes 2	9_104118330_A_G	ENST00000374793.8:c.1951A>G	p.Thr651Ala^d^	26.1	8.5	0.96			Cell cycle; mitosis
F36	FANCI	FA complementation group I	15_89295009_C_T	ENST00000310775.12:c.2551C>T	Gln851Ter	43		0.96	0.48	NA	Cell cycle; DNA repair
F36	MCPH1	Microcephalin 1	8_6442069_T_TC	ENST00000344683.10:c.586dup	Gln196ProfsTer8	25.5		1.34	0.32	Iron binding (*p* = 7.1352e‐05); Catalytic site (*p* = 0.0006); Disulfide linkage (*p* = 0.00156); Proteolytic cleavage (*p* = 0.005); Signal helix (*p* = 0.0055)	Cell cycle; Chromosome condensation and DNA damage response
F43	ENDOV	Endonuclease V	17_80425035_C_T	ENST00000518137.6/c.520C>T	p.Arg174Ter	35		1.44	0.42	NA	DNA repair
F1_S3	ZW10	Zw10 Kinetochore Protein	11‐113,736,645‐C‐G	ENST00000200135.8:c.2194G>C	p.Ala732Pro^d^	27	5.5	1.53			Cell cycle; mitosis
F12_S2	EXO1	Exonuclease 1	1_241889544_G_T	ENST00000366548.8:c.2485G>T	Glu829Ter	45		1.01	0.5	NA	Cell cycle; DNA repair
F12_S2	RECQL5	RecQ Like Helicase 5	17_75660989_T_C	ENST00000317905.10:c.952A>G	p.Ser318Gly^d^	27.3	6.75	0.6			Cell cycle; DNA damage response
F12_S2	XRCC1	X‐Ray Repair Cross Complementing 1	19_43554680_C_T	ENST00000262887.10:c.380G>A	p.Arg127Gln	28	5	0.56			Base excision repair
ECM, Focal adhesion, TGFβ signaling											
F6	LAMC2	Laminin Subunit Gamma 2	1_183227656_C_T	ENST00000264144.5:c.1427C>T	p.Thr476Met	23.7	6.5	1.35			ECM organization; Focal adhesion
F6	SMAD4	SMAD Family Member 4	18_51058218_CTGGT_C	ENST00000342988.8:c.762‐765del	Gly255SerfsTer80	29.5		0.17	0.69	NA	Tumor suppressor; TGFbeta signaling
F7	COL12A1	Collagen Type XII Alpha 1 Chain	6_75102661_G_T	ENST00000322507.13:c.8351C>A	p.Pro2784His^d^	27	7	3.55			ECM organization
F8	COL4A4	Collagen Type IV Alpha 4 Chain	2_227007353_C_T	ENST00000396625.5:c.5045G>A	p.Arg1682Gln^d^	27.1	7	2.22			ECM organization; Focal adhesion
F8	SMAD6	SMAD Family Member 6	15_66703558_TGGGAGCTCCCTGCTGGACGTGGCGGAGCC_T	ENST00000288840.10:c.306_334del	Ser102ArgfsTer9	28.3		2	0.53	NA	BMP and TGF‐beta/activin‐signaling
F34	ADAMTS14	ADAM Metallopeptidase With Thrombospondin Type 1 Motif 14	10_70752124_C_T	ENST00000373207.2:c.2626C>T	p.Arg876Cys	29.7	6.5	0.79			ECM organization
F34	ACVR1C	Activin A Receptor Type 1C	2_157556276_G_C	ENST00000243349.13:c.361C>G	p.Pro121Ala	21.5	6	1.96			TGFbeta signaling
F36	COL1A2	Collagen Type I Alpha 2 Chain	7_94427840_C_T	ENST00000297268.11:c.3481C>T	p.Arg1161Cys^d^	29.5	7	3.53			ECM organization; Focal adhesion
F36	ITGB4	Integrin Subunit Beta 4	17_75756515_C_T	ENST00000200181.8:c.4795C>T	p.Arg1599Cys^d^	31	5.5	2.17			ECM organization; Focal adhesion
F43	COL6A3	Collagen Type VI Alpha 3 Chain	2_237365895_G_A	ENST00000295550.9:c.5641C>T	p.Arg1881Cys	24.6	5	2.09			ECM organization; Focal adhesion
F1_S3	FLNC	Filamin C	7_128840655_G_A	ENST00000325888.13:c.1657G>A	p.Gly553Ser^d^	29.8	8.5	5.95			Focal adhesion
F1_S3	ITGA10	Integrin Subunit Alpha 10	1_145897010_C_A	ENST00000369304.8:c.2744+1G>T^c^		24.5		0.83			ECM organization; Focal adhesion
F12_S2	COL11A1	Collagen Type XI Alpha 1 Chain	1_102995999_C_T	ENST00000370096.9:c.2285G>A	p.Arg762Gln^d^	28.1	8	1			ECM organization
Candidate tumor suppressor gene for CRC											
F6	DMBT1	Deleted In Malignant Brain Tumors 1	10_122589066_G_A	ENST00000338354.10:c.1906G>A	p.Ala636Thr^d^	24.8	5	−0.37			Candidate TSG for CRC; Immune response
F34	DMBT1	Deleted in malignant brain tumors 1	10_122589000_C_T	ENST00000338354.10:c.1840C>T	Arg614Ter	33		0.91	0.5	Manganese binding (*p* = 0.001); Disulfide linkage (*p* = 0.003); RNA binding (*p* = 0.005); Cadmium binding (*p* = 0.005); Iron binding (*p* = 0.005)	Candidate TSG for CRC; Immune response

^a^ gnomAD missense score (*Z* positive → increased constraint); gnomAD LoF score (LOEUF = loss‐of‐function observed/expected upper bound fraction; <0.6 for Mendelian diseases).

^b^ SpliceAI‐donor‐loss Δ score = 1; MMSp_donor score −4.034.

^c^ SpliceAI‐donor‐loss Δ score = 0.94; MMSp_donor score −6.955.

^d^ Pathogenicity of the missense variants was supported by the AlphaMissense and/or REVEL predictions.

Among the “Cell cycle” genes were *CHEK2*, *EXO1*, *FANCI*, *MCPH1*, *PRC1*, *RECQL5* and *SMC2* from our list. From these, *FANCI* (stop‐gain) and *MCPH1* (frameshift) variants were present in family F36. The MutPred LOF‐scores of these variants were less than the recommended threshold of 0.5, but the MCPH1 variant was predicted to affect iron binding, catalytic site, disulfide linkage, proteolytic cleavage and signal helix. Details of the identified variants in these genes are shown in Table 2. Although STRING did not connect *ENDOV* (family F43, stop‐gain, CADD 35), *ZW10* (family F1_S3, missense, CADD 27) and *CDK18* (family F29, splice site, CADD 34) in the DNA repair/cell cycle cluster, these are related genes (Table 2).

Another large network included 14 genes from three different STRING clusters related to KEGG pathways “Focal adhesion” (FDR 0.0063), “Extracellular matrix‐receptor interaction” (FDR 0.0288) and Reactome pathway “Degradation of extracellular matrix” (FDR 0.0361) (Table 1). From these, the *ITGA10* splice site variant, which was predicted to lead to the loss of donor site in intron 22, was the most interesting one. Also, four genes from KEGG pathway “TGFβ signaling” were among the 14 genes in this network. In addition to the *SMAD4* frameshift variant in family F6 described above, a frameshift variant with a MutPred LOF‐score of 0.53 in the *SMAD6* was detected in family F8. Details of the identified variants in this network are shown in Table 2.

## 4 DISCUSSION

Studies on germline genetics of cancer have applied various approaches ranging from linkage to genome‐wide association studies (GWASs) based on the different underlining assumptions. The assumption in linkage studies is that as biological relatives share part of their genomes they do share also the harmful variants which may be the cause of their disease. Until year 2000 linkage approach was able to deliver the largest number of cancer susceptibility genes.^40^ GWASs use large numbers of patients and compare their genetic linkage distributions to those of healthy controls.^41^ They have been very productive but for many of the detected loci no function can be assigned. Here we applied a linkage‐type of approach but instead of large affected families (which are difficult to locate) we focused on nine CRC families with the index patient presenting with a double primary CRC known to signal high familial/individual risk.^12^

As an important technical note, the patients were screened at the diagnostic setting for the most common expected mutations using methodologies available at the time of diagnosis, 3 patients with next‐generation sequencing (NGS) high risk panel (19 genes), the two previously reported families with a smaller panel of 8 genes or specific mutations, 3 patients for MMR founder mutations using Multiplex Ligation‐dependent Probe Amplification (MLPA) and one patient (family F6) by DHPLC, which apparently missed the *CHEK2* variant. In our present WES approach, the detected variants throughout the exome were subjected to a stringent filtering. Only the top 1% of potentially deleterious, rare (MAF <0.1%) variants, according to the CADD tool, were selected for a manual inspection of their pathogenic potential. The in silico tools used gave evidence on the effect of the amino acid change on the protein function, their location at the evolutionary conserved position and the intolerance of the corresponding genes to variation. As a final step protein–protein interaction between the prioritized genes and the well‐known CRC predisposition genes was studied in an attempt to strengthen the connection of our genes to relevant pathways in CRC development.

In the exome of the nine CRC patients with a personal and family history of CRC we identified potential CRC predisposition variants in genes involved in DNA repair, cell cycle, TGFβ signaling and extracellular matrix related functions. However, only in one gene, *DMBT1*, two different variants were found in two different families, a missense variant Ala636Thr in family F6 and a stop‐gain variant Arg614Ter in family F34. The role of this gene in CRC has remained elusive but it has been suggested to mediate inflammatory responses and to be related to inflammatory bowel disease.^42^ In family F6, also two variants in known CRC predisposition genes were detected, including *CHEK2* and *SMAD4*, in addition to several likely candidates.

The finding of a *CHEK2* variant in a patient with a double primary CRC may not be a coincidence because among the 28 CRC families with a family member diagnosed with a double primary CRC, that we reviewed at the International Hereditary Cancer Center in Szczecin, Poland, two had previously been diagnosed with the *CHEK2* variant. As such, double primaries are not very rare and they are diagnosed in a few percent of CRC patients, more commonly in Lynch syndrome and much more commonly in familial adenomatous polyposis patients.^43^ *CHEK2* Ile157Thr mutation has been detected in 7% of Polish CRC patients and in 5% of controls conferring a 1.5‐fold increased risk of CRC.^35^ Due to its high frequency in the Polish population, the variant alone may not confer the strong aggregation of cancer in family F6, but it may act as a modifier of another variant, such as the frameshift variant in *SMAD4*, also found in this family. Unfortunately, we did not have access to other samples in the family for a segregation analysis.

Using the STRING PPI network and pathway analysis we identified a cluster of 20 proteins, including DNA repair/cell cycle proteins which included products of genes *CHEK2*, *EXO1*, *FAAP24*, *FANCI*, *MCPH1*, *POLL*, *PRC1*, *RECQL*, *RECQL5*, *SMC2* and *XRCC1*. In addition, *RRM2* and *SHCBP1* were clustered together with these genes. Other interesting genes, not clustered together with the above genes, but related to DNA repair/cell cycle included *CDK18*, *ENDOV* and *ZW10*. As pointed out above, *CHEK2* was detected in family F6, and other DNA repair/cell cycle genes that were detected in single families were *PRC1*, *ENDOV* and *ZW10*, found in families F8, F43 and in the previously reported family F1_S3,^5^ respectively. Variants in *FANCI* and *MCPH1* were present in family F36, those in *SMC2* and *FAAP24* were present in family F34 and those in *RRM2* and *CDK18* in family 29. In two families, three DNA repair/cell cycle gene variants were found, including family F7 (*POLL*, *RECQL*, *SHCBP1*) and the previously reported family F12_S2 (*EXO1*, *RECQL5*, *XRCC1*).^6^

Some previous WES studies have identified candidate genes for CRC susceptibility,^1^ and for example RECQL from our list was reported among the most promising candidate genes for germline CRC predisposition in a Spanish study.^44^ Large collection of DNA repair genes have been analyzed in terms of their association with chromosomal aberrations, and for example RRM2B, a ribonucleotide reductase related to our candidate gene RRM2, was associated with chromosomal aberrations.^45^

The other large STRING network included extracellular matrix and TGFβ signaling genes, the latter of which included *SMAD4* and *SMAD6*. A frameshift variant in *SMAD4* was described above in family F6 and another frameshift variant in *SMAD6* was found in family F8. In colonic epithelial cells, TGFβ signaling promotes differentiation and apoptosis, and disruption of the signaling leads to tumor progression through epithelial transformation and tumor‐stromal interactions in a cascade of epithelial‐mesenchymal transition (EMT).^46^, ^47^ Mutations were found in collagen and integrin genes and these may be related to TGFβ signaling related tumor‐stromal interactions in EMT.^48^

The prioritization of the variants in our study was based on a set of in silico tools, which can only offer predictions. Also, we did not have any samples from the other CRC cases of the families to do a segregation analysis, which could support the pathogenicity of the variants and their co‐segregation in the diseased individuals. We acknowledge that a conclusive classification of the variants is only possible with the help of functional tests and analysis of the segregation of the variants with the disease in the affected families. Therefore, further studies will be required to determine the potentially synergistic contribution of the variants in familial CRC. However, our present study had a unique structure of sequencing one high‐risk patient with a double primary CRC from families of multiple CRC patients. The case number was only nine, which was an obvious limitation, and indeed mutations in only one likely susceptibility gene, *DMBT1*, were found in two families, one of which was family F6. However, the index individual from this family harbored mutations also in *CHEK2* and *SMAD4*, known susceptibility genes for CRC. *SMAD4* is one of the commonly mutated somatic drivers of CRC^49^ and germline mutations in *SMAD4* predispose to juvenile polyposis syndrome.^1^ Unfortunately, we did not have any phenotypic or clinical information about the patient to confirm the diagnosis, nor did we have any other samples from the family to do a segregation analysis. Among the eight other index patients, five harbored at least two candidate DNA repair/cell cycle variants which fulfilled our filtering criteria and two a candidate gene from both the DNA repair/cell cycle and TGFβ and extracellular matrix cluster each. We did not do any general confirmation of the variants by Sanger sequencing, which is another limitation of our study.

Although we cannot define the contribution of the indicated variants to CRCs in the index patients or their families, the known overwhelming role of DNA repair/cell cycle genes in familial CRC provides circumstantial evidence of suggestive role of the indicated genes. Our results support the observation of several other recent studies that the novel high‐to‐moderate penetrance variants may be private to a family, and they may follow a polygenic mode of inheritance.^1^, ^44^ The confirmation requires a thorough genomic and functional analysis combined with a variant segregation analysis in the affected family to verify the pathogenicity of each variant.

## AUTHOR CONTRIBUTIONS

**Asta Försti:** Conceptualization; investigation; writing – original draft; methodology; visualization; writing – review and editing; formal analysis; project administration; supervision; data curation. **Filip Ambrozkiewicz:** Investigation; writing – review and editing; formal analysis. **Magdalena Marciniak:** Validation; writing – review and editing; resources; investigation; data curation. **Jan Lubinski:** Writing – review and editing; resources; data curation; conceptualization. **Kari Hemminki:** Conceptualization; funding acquisition; writing – original draft; writing – review and editing.

## FUNDING INFORMATION

Czech ministry grants NU21‐03‐00506, NU21‐03‐00145 and NW24‐03‐00521; Czech Science Agency grant 23‐05609S; the project National Institute for Cancer Research—NICR (Programme EXCELES, ID Project No. LX22NPO5102), funded by the European Union.

## CONFLICT OF INTEREST STATEMENT

The authors declare no conflicts of interest.

## ETHICS STATEMENT

Collection of patient samples and associated clinical information was undertaken with written informed consent in accordance with the tenets of the Declaration of Helsinki. The study was approved by the Bioethics Committee of the Pomeranian Medical University in Szczecin (protocol code No: BN‐001/174/05).

## Supporting information


**Data S1.** Supporting Information.

**Table S3.** (A) Overview of the missense variants prioritized in cancer patients with personal and family history of CRC. Variants potentially affecting protein functions relevant to CRC development are highlighted in bold. (B) Overview of the stop‐gain variants leading to truncated proteins in cancer patients with personal and family history of CRC. Variants potentially affecting protein functions relevant to CRC development are highlighted in bold. (C) Overview of the canonical splice‐site variants in cancer patients with personal and family history of CRC. Variants potentially affecting protein functions relevant to CRC development are highlighted in bold. (D) Overview of the frame‐shift variants leading to truncated proteins in cancer patients with personal and family history of CRC. Variants potentially affecting protein functions relevant to CRC development are highlighted in bold.

## Data Availability

The WES data generated in this study are available in the European Genome‐Phenome Archive (EGA) under accession number EGAS50000000606 and EGAS00001005118.
