# Supplementary material for: Search for germline gene variants in colorectal cancer families presenting with multiple primary colorectal cancers
Source: Int J Cancer. 2024 Dec 10;156(7):1393–403. doi: 10.1002/ijc.35283 (PMC11789446; doi:10.1002/ijc.35283)
Supplement: Supplementary file 1 — Data S1. Supporting Information. [file IJC-156-1393-s001.pdf]

SEARCH FOR GERMLINE GENE VARIANTS IN COLORECTAL CANCER FAMILIES PRESENTING WITH MULTIPLE PRIMARY COLORECTAL CANCERS

Asta Försti, Filip Ambrozkiewicz, Magdalena Marciniak, Jan Lubinski and Kari Hemminki

Table of contents

Supplementary Table 1. Colorectal cancer (CRC) families with one family member diagnosed with at least two CRCs. CRC and other cancer cases in each family are shown as well as the method used previously to identify a pathogenic mutation in each family and the mutation(s) detected. Families selected for whole-exome sequencing are shown in bold. 1°/2°/3°, first/second/third degree relative; CSU, cancer site unknown; NGS, next generation sequencing; DHPLC, denaturing high performance liquid chromatography; MLPA, multiplex ligation-dependent probe amplification.

Supplementary Table 2. Summary of the sequencing coverage and quality statistics for each sample.

Supplementary Table 3. Available as a separate Excel file. (A) Overview of the missense variants prioritized in cancer patients with personal and family history of CRC. Variants potentially affecting protein functions relevant to CRC development are highlighted in bold. **(B)** Overview of the stop-gain variants leading to truncated proteins in cancer patients with personal and family history of CRC. Variants potentially affecting protein functions relevant to CRC development are highlighted in bold. **(C)** Overview of the canonical splice-site variants in cancer patients with personal and family history of CRC. Variants potentially affecting protein functions relevant to CRC development are highlighted in bold. **(D)** Overview of the frame-shift variants leading to truncated proteins in cancer patients with personal and family history of CRC. Variants potentially affecting protein functions relevant to CRC development are highlighted in bold.

Supplementary Figure 1. Pedigrees of the nine families. Index case subjected to whole-exome sequencing is shown by *. CRC in the families are shown in black symbols, all other cancer cases in light grey symbols.

Supplementary table 1. Colorectal cancer (CRC) families with one family member diagnosed with at least two CRCs. CRC and other cancer cases in each family are shown as well as the method used previously to identify a pathogenic mutation in each family and the mutation(s) detected. Families selected for whole-exome sequencing are shown in bold. 1°/2°/3°, first/second/third degree relative; CSU, cancer site unknown; NGS, next generation sequencing; DHPLC, denaturing high performance liquid chromatography; MLPA, multiplex ligation-dependent probe amplification.

Family	CRC cases	Other cancers	Method	Genes	Detected mutation
**F1**	Colon 43y+70y; Colon 82y (1°); Colon (1°)	Stomach 42y (2°); CSU 80y (2°); Stomach (1°)	NGS (HiRisk panel)	APC, ATM, BRCA1, BRCA2, CDH1, CDKN2A, CHEK2, MLH1, MUTYH, MSH2, MSH6, NBN, PALB2, PTEN, PMS2, RAD51C, RAD51D, STK11, TP53	MUTYH c.1465G>T (VUS)
**F5**	Colon 46y+64y		NGS (HiRisk panel)	APC, ATM, BRCA1, BRCA2, CDH1, CDKN2A, CHEK2, MLH1, MUTYH, MSH2, MSH6, NBN, PALB2, PTEN, PMS2, RAD51C, RAD51D, STK11, TP53	CHEK2 c.470C>T (missense), ATM c.1342C>T (VUS)
**F6**	**Colon 28y+53y; Colon 63y (1°); Colon 43y (1°); Colon (2°)**	**Endometrial 25y (3°); Laryngeal 19y (3°)**	**DHPLC**		
**F7**	**Colon 64y+71y; Colon 47y (1°)**		**NGS (HiRisk panel)**	**APC, ATM, BRCA1, BRCA2, CDH1, CDKN2A, CHEK2, MLH1, MUTYH, MSH2, MSH6, NBN, PALB2, PTEN, PMS2, RAD51C, RAD51D, STK11, TP53**	
**F8**	**Colon 64y+71y; Colon 47y (1°)**		**NGS (HiRisk panel)**	**APC, ATM, BRCA1, BRCA2, CDH1, CDKN2A, CHEK2, MLH1, MUTYH, MSH2, MSH6, NBN, PALB2, PTEN, PMS2, RAD51C, RAD51D, STK11, TP53**	
**F10**	Colon 51y+55y +Kidney 60y	CSU (1°); CSU 63y (1°); CSU ~50y (1°)	DHPLC, MLPA		
**F11**	Colon 72y+73y				
**F12**	Colon 54y+68y	Lung 90y (1°)	MMR founder mutations, MLPA		
**F14**	Colon 69y+71y		MMR founder mutations, MLPA		
**F15**	Colon 49y+58y +Colon polyp 64y	CSU ~62y (2°)	NGS (HiRisk panel)	APC, ATM, BRCA1, BRCA2, CDH1, CDKN2A, CHEK2, MLH1, MUTYH, MSH2, MSH6, NBN, PALB2, PTEN, PMS2, RAD51C, RAD51D, STK11, TP53	

**F17**	Colon 71y+72y	Brain 60y (1°)	MMR founder mutations, MLPA		
**F18**	Colon 56y+59y; Colon 80y (2°); Colon polyp 56y (1°)		MMR founder mutations, MLPA		
**F19**	Colon 61y+67y		MMR founder mutations, MLPA		
**F22**	Colon 41y+44y	CSU 21y (1°)	NGS (HiRisk panel)	APC, ATM, BRCA1, BRCA2, CDH1, CDKN2A, CHEK2, MLH1, MUTYH, MSH2, MSH6, NBN, PALB2, PTEN, PMS2, RAD51C, RAD51D, STK11, TP53	
**F23**	Colon 53y+55y +Breast 56y	Lung (2°)	MMR founder mutations, MLPA		
**F24**	Colon 52y+55y; Colon polyp 77y (1°); Colon polyp 44y (1°)	Stomach 57y (1°)	MMR founder mutations, MLPA		
**F26**	Colon 48y+51y Colon polyp 47y (1°)		NGS (HiRisk panel)	APC, ATM, BRCA1, BRCA2, CDH1, CDKN2A, CHEK2, MLH1, MUTYH, MSH2, MSH6, NBN, PALB2, PTEN, PMS2, RAD51C, RAD51D, STK11, TP53	
**F27**	Colon 55y+55y		MMR founder mutations, MLPA		
**F29**	**Colon 60y+61y; Colon 56y (1°)**		**MMR founder mutations, MLPA**		
**F31**	Colon 73y+74y	CSU (1°); Prostate (1°)	MMR founder mutations, MLPA		
**F34**	**Colon 64y+64y; Colon 80y (1°); Colon 66y (1°)**	**Lung <64y (1°); CSU <56y ((1°)**	**MMR founder mutations, MLPA**		

**F36**	**Colon 61y+63y; Colon <56y (1°); Colon <53y (1°); Colon 47y (1°)**	**Pancreas 50y (1°)**	**NGS (HiRisk panel)**	**APC, ATM, BRCA1, BRCA2, CDH1, CDKN2A, CHEK2, MLH1, MUTYH, MSH2, MSH6, NBN, PALB2, PTEN, PMS2, RAD51C, RAD51D, STK11, TP53**	
**F38**	Colon 54y+71y		MMR founder mutations, MLPA		
**F39**	Colon 84y+85y; Colon 55y (2°)	CSU (1°); CSU (1°)	MMR founder mutations, MLPA		
**F41**	Colon 39y+44y; Colon 54y (1°)		NGS (HiRisk panel)	APC, ATM, BRCA1, BRCA2, CDH1, CDKN2A, CHEK2, MLH1, MUTYH, MSH2, MSH6, NBN, PALB2, PTEN, PMS2, RAD51C, RAD51D, STK11, TP53	CHEK2 c.470C>T (missense)
**F43**	**Colon 68y+80y; Colon <60y (1°)**	**Ovarian 45y (2°)**	**MMR founder mutations, MLPA**		
**F1_S3***	**Colon 52y+52y; Colon 35y (1°); Colon 48y (1°); Colon 83y (2°); Colon (2°); Colon (2°); Colon 61y (3°); Colon 70y (3°); Colon polyps 56y+59y+71y (1°)**			**APC, MLH1, MSH2, MSH3, large deletions in EPCAM, POLE p.Leu424Val, POLD1 p.Ser478Asn, NTHL1 p.Gln90***	
**F12_S2#**	**Colon 56y+63y; Colon 78y (1°); Colon 65y (1°)**			**APC, MLH1, MSH2, MSH3, large deletions in EPCAM, POLE p.Leu424Val, POLD1 p.Ser478Asn, NTHL1 p.Gln90***	

* Reported in Skopelitou D. et. al. Int. J. Mol. Sci. 2021 (ref. 5 in the manuscript)

Reported in Zhu L. et. al. Cancers (Basel) 2022 (ref. 6 in the manuscript)

Supplementary Table S2. Summary of the sequencing coverage and quality statistics for each sample.

Sample ID	Total number of sequenced reads	Total number of uniquely mapped non-duplicate reads	Total number of covered bases	Average coverage per base	Percentage of targeted bases with coverage ≥20	Library preparation kit	Sequencing platform
**F6**	87600000	86213240	12931986000	118.59	97.99	Agilent_V6	DNBSEQ
**F7**	88000000	86210440	12931566000	124.76	98.16	Agilent_V6	DNBSEQ
**F8**	88800000	86181010	12927151500	124.71	98	Agilent_V6	DNBSEQ
**F29**	89200000	86232772	12934915800	124.57	98.06	Agilent_V6	DNBSEQ
**F34**	87600000	86118656	12917798400	122.18	98.24	Agilent_V6	DNBSEQ
**F36**	88400000	86169042	12925356300	120.46	98.18	Agilent_V6	DNBSEQ
**F43**	90000000	86236624	12935493600	110.73	97.65	Agilent_V6	DNBSEQ
**F1_S3***	132920042	124896092	NA	60.98	71**	Ag SuSe V5+UTRs	Illumina HiSeq 2000
**F12_S2***	1044409207	908635792	NA	40.72	98**	Illumina TruSeq Nano DNA	Illumina HiSeq X Ten V2.5

*As reported in Zhu L, et.al. Germline Variants of CYBA and TRPM4 Predispose to Familial Colorectal Cancer. Cancers (Basel). 14(3):670. doi:10.3390/cancers14030670

**Percentage of targeted bases with coverage ≥10

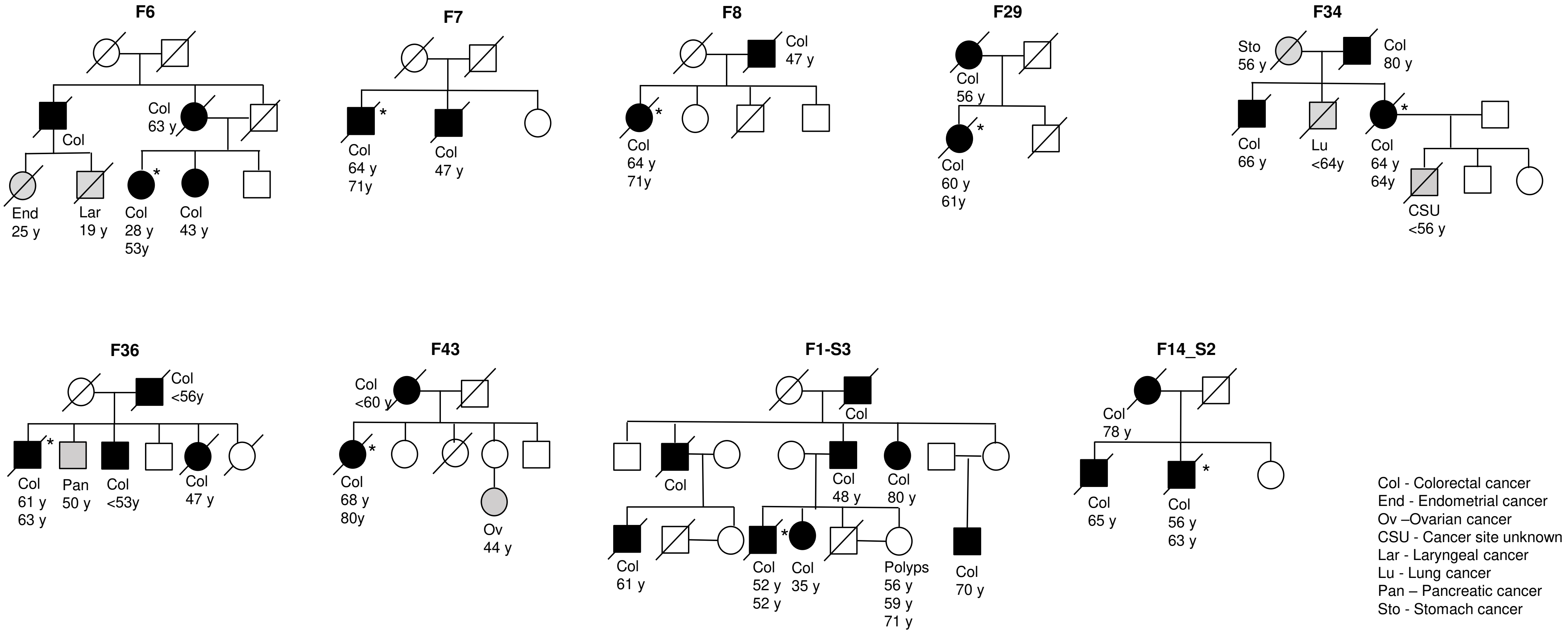

Supplementary Figure 1. Pedigrees of the nine families. Index case subjected to whole-exome sequencing is shown by *. Colorectal cancers in the families are shown in black symbols, all other cancer cases in light grey symbols.
